# Engineering Nano-Sized Silicon Anodes with Conductive Networks toward a High Average Coulombic Efficiency of 90.2% via Plasma-Assisted Milling

**DOI:** 10.3390/nano14080660

**Published:** 2024-04-10

**Authors:** Yezhan Zuo, Xingyu Xiong, Zhenzhong Yang, Yihui Sang, Haolin Zhang, Fanbo Meng, Renzong Hu

**Affiliations:** 1 Guangdong Provincial Key Laboratory of Advanced Energy Storage Materials, School of Materials Science and Engineering, South China University of Technology, Guangzhou 510640, China; 2 School of Energy and Mechanical Engineering, Nanjing Normal University, Nanjing 210023, China

**Keywords:** plasma-assisted milling, initial coulombic efficiency, silicon anode, structural engineering, lithium-ion battery

## Abstract

Si-based anode is considered one of the ideal anodes for high energy density lithium-ion batteries due to its high theoretical capacity of 4200 mAh g^−1^. To accelerate the commercial progress of Si material, the multi-issue of extreme volume expansion and low intrinsic electronic conductivity needs to be settled. Herein, a series of nano-sized Si particles with conductive networks are synthesized via the dielectric barrier discharge plasma (DBDP) assisted milling. The p-milling method can effectively refine the particle sizes of pristine Si without destroying its crystal structure, resulting in large Brunauer–Emmett–Teller (BET) values with more active sites for Li^+^ ions. Due to their unique structure and flexibility, CNTs can be uniformly distributed among the Si particles and the prepared Si electrodes exhibit better structural stability during the continuous lithiation/de-lithiation process. Moreover, the CNT network accelerates the transport of ions and electrons in the Si particles. As a result, the nano-sized Si anodes with CNTs conductive network can deliver an extremely high average initial Coulombic efficiency (ICE) reach of 90.2% with enhanced cyclic property and rate capability. The C-PMSi-50:1 anode presents 615 mAh g^−1^ after 100 cycles and 979 mAh g^−1^ under the current density of 5 A g^−1^. Moreover, the manufactured Si||LiNi_0.8_Co_0.1_Mn_0.1_O_2_ pouch cell maintains a high ICE of >85%. This work may supply a new insight for designing the nano-sized Si and further promoting its commercial applications.

## 1. Introduction

Lithium-ion batteries (LIBs) have progressively dominated the market of consumer electronics, electric vehicles (EVs), and energy storage grids in the last 30 years [1,2,3] because of their environmentally friendly nature, high energy density, and long lifespan [4,5]. Although the industrial processing technology has improved, the current commercialized graphite anode is insufficient to meet the market demand of high energy density due to its limited capacity of 372 mAh g^−1^ [6], especially for the increasing EV market. The demand for LIBs of EVs has accounted for >50% of the total demand since 2020, which has continued to increase over the years [7]. According to The World Economic Forum prediction, the global battery demand will be 2600 GWh in 2030 and the demand will reach approximately 5500 GWh in 2040 by the conservative prediction model [8]. Among various anode materials, Si-based anodes have attracted attention due to the high theoretical capacity of 4200 mAh g^−1^ and moderate operation voltage of <0.4 V vs. Li/Li^+^ [9,10]. However, the commercial applications of Si-based anodes are basically hindered by their extreme volume expansion of >300% during the lithiation/de-lithiation process and low intrinsic electronic conductivity [11,12,13].

On the one hand, the large volume expansion would cause severe particle pulverization, poor electric contact, and unstable solid electrolyte interphase (SEI) [9,14]. These problems dramatically reduce the cyclic property of LIBs. To alleviate the volume expansion, novel Si particle structural designs have been proposed, such as nanostructure [15,16,17], core-/york-shell structure [18,19,20], and porous structure [21,22]. For nanostructured materials, the nanomechanical properties at specific local regions could enhance the overall performance of the material [23,24]. The local stress changes caused by volume expansion in nano-Si anode can be effectively alleviated. Ball milling is a simple and low-cost strategy for mass production of nanomaterials [25]. The particles can be effectively refined by introducing a plasma in the milling process owing to the synergistic effect of the instant heating of the plasma, the electron collision in the electric field, and the mechanical impact of the milling [26]. In our previous study, we prepared a series of nano-materials for LIB electrodes with good properties by dielectric barrier discharge plasma (DBDP)-assisted milling (abbreviated as P-milling) [27,28]. On the other hand, the low electronic conductivity of Si and the thin oxide layer formed on Si particles would result in poor electrochemical kinetics during the Li^+^ alloying/dealloying process, causing low initial Coulombic efficiency (ICE) [29,30]. Recently, single-walled carbon nanotubes (CNTs) have been reported as addictive to improve the electronic conductivity of Si-based anodes by forming a conductive network [31,32]. Moreover, the long and thin CNTs are hard to disperse due to their unique structure, which results in strong van der Waals forces between nanotubes [31]. However, such flexibility of CNTs and their strong interaction characteristic can be applied to strengthen the electrical contact among Si particles in electrodes, which would eventually improve the cyclic property of Si-based anodes, even without conventional conductive or binders [31,32].

In this work, P-milling was applied to prepare the nano-sized Si particles by controlling the mass ratio of balls to material, i.e., 10:1, 20:1, 50:1, and 100:1, respectively. To simplify the description and comparison in this article, the mass ratio of balls to material is used to represent the corresponding powder and anode samples. By adding additional CNTs and aqueous dispersion in the conventional slurry-coating process, we prepared the P-milling Si composite anodes with a CNT conductive network (C-PMSi anodes). Moreover, pristine Si and P-milling Si anodes (PMSi anodes) were manufactured by the same process without CNTs for comparison. Based on a series of electrochemical measurements and structural characterizations, it is found that C-PMSi anodes present higher ICEs, better capacity retention, and lower resistance. Particularly, the C-PMSi-50:1 electrode exhibits the lowest volume expansion and intact electrode after cycling processes. Moreover, C-PMSi/G||Li and C-PMSi/G-P||LiNi_0.8_Co_0.1_Mn_0.1_O_2_ (NCM811) pouch cells are also prepared to investigate practical applications and both batteries exhibited high ICEs of 89.0% and 85.3%.

## 2. Materials and Methods

### 2.1. Material Preparation

The commercial Si powder (99.9% pure, 1 μm, ST-NANO Science and Technology Co., Ltd., Shanghai, China) was used as a precursor material without further purification. The milling balls were sealed in the milling vial together with the Si powder in a mass ratio of 10:1, 20:1, 50:1, and 100:1, respectively. The milling balls contain two diameters of 12 mm and 8 mm, for a mass ratio of 1:1. For all processes, the milling vial was sealed in an Ar-filled glove box (H_2_O, O_2_ < 0.01 ppm) and P-milling was conducted under pure Ar atmosphere (0.1 MPa) with a vibration type ball milling machine.

PMSi/G was blended by 20 wt. % PMSi-50:1 and 80 wt. % graphite (99.9%, pure, BASF Shanshan Battery Materials Co., Ltd., Changsha, China) powder for 4 h through high energy milling (C-PMSi/G-B) and the Ar-assisted plasma (C-PMSi/G-P) process, respectively. High energy milling was conducted as P-milling in a mass ratio of 50:1 without Ar plasma. The Ar-assisted plasma process was conducted as P-milling without balls.

### 2.2. Material Characterization

The phase structure of powder samples was characterized by an X-ray diffractometer (XRD, Panalytical) with Cu Kα radiation. The specific surface areas of samples were determined by using the BET method with a gas reaction controller (Autosorb iQ) by nitrogen absorption and each sample volume occupied one-third of the sample tube. The morphology and microstructure of powder and electrode samples were observed by using field emission scanning electron microscopy (SEM, TESCAN GAIA3). The acceleration voltage of SEM is settled to 5 kV and no additional contrast agent was performed on the samples.

### 2.3. Electrochemical Measurement

The electrochemical measurements of the half-cells (CR2016-type coin cell) and full-cells (pouch cell) were assembled in an Ar-filled glove box (H_2_O, O_2_ < 0.01 ppm). The electrodes are separated by separators (Celgrad 2325) in the cells. The electrolyte is 1 M LiPF_6_ in a mixture of ethylene carbonate (EC) and diethyl carbonate (DEC) with a volume ratio of 1:2, with 10 wt. % fluoroethylene carbonate (FEC). The PMSi electrodes were prepared by mixing 80 wt. % of the various active materials, 10 wt. % of conductive agent (Super C45), and 10 wt. % binder (carboxymethyl cellulose, CMC) into a uniform slurry. The slurry was then coated onto Cu foil and dried at 80 °C for 12 h under vacuum. Similarly, the C-PMSi electrodes were prepared via the above step by adding additional CNT aqueous dispersion (0.4 wt. % SWCNT in water, 0.6. wt. % CMC as a surfactant stabilizer, provided by Zhuhai CosMX Battery Co., Ltd., Zhuhai, China) into the slurry. The mass ratio of mixing powders to CNTs dispersion is 1:1 and the content of CNTs in the electrode is 0.396 wt. % by calculation. The average mass loading of the PMSi and C-PMSi electrodes was ~1.5 mg cm^−2^. The NCM811 electrodes were also prepared by the slurry-coating method, consisted of 80 wt. % NCM811 powders (99.9%, pure, Canrd New Energy Technology Co., Ltd., Dongguan, China), 10 wt. % Super C45, and 10 wt. % binder (polyvinylidene difluoride, PVDF) coating and drying on Al foil, with an average mass loading of ~5.2 mg cm^−2^. The galvanostatic charge/discharge test and rate test were conducted by a multichannel battery test system (LANHE, Wuhan, China, LAND-CT2001A) at room temperature. The galvanostatic voltage range was 0.01~1.5 V vs. Li/Li^+^. The specific capacity was calculated based on the total mass of active material without conductivity agent and binder. Cyclic voltammogram (CV) measurements were conducted on an electrochemical station (Gamry, Warminster, PA, USA, Interface 1000) at a scan rate of 0.1 mV s^−1^. Electrochemical impedance spectroscopy (EIS) measurements were performed on an electrochemical station (Gamry, Warminster, PA, USA, Interface 1000) in the frequency range from 1 MHz to 0.01 Hz.

## 3. Results and Discussion

**Figure 1:**
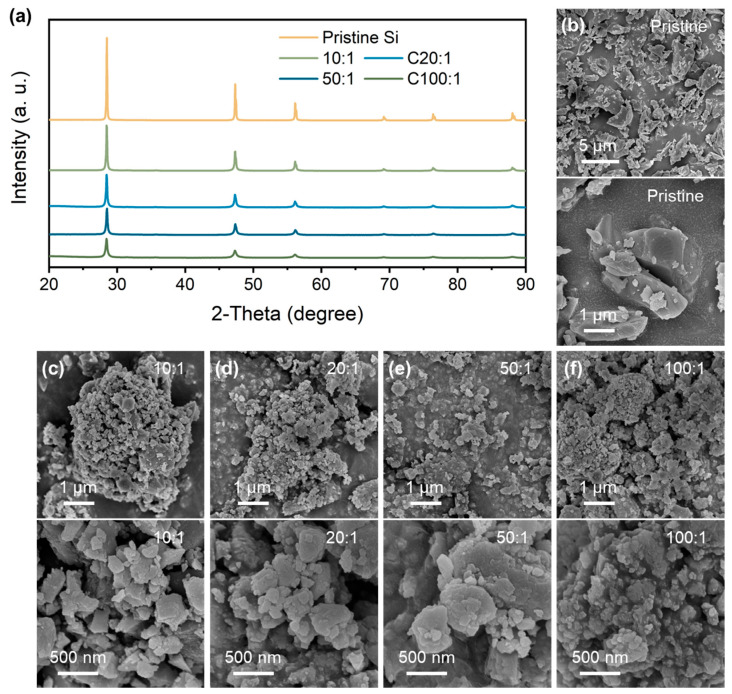
(**a**) XRD patterns of pristine Si and P-milling samples. SEM and HRSEM images of (**b**) pristine Si and P-milling particles with a mass ratio of (**c**) 10:1, (**d**) 20:1, (**e**) 50:1, and (**f**) 100:1.

Figure 1a shows the XRD patterns of the pristine Si and P-milling powders prepared by the P-milling method. It is shown that the diffraction peaks located at 28.48°, 47.33°, and 56.15° are attributed to the (1 1 1), (2 2 0), and (3 1 1) plane of the Si materials. With the mass ratio increasing from 10:1 to 100:1, P-milling samples show significantly weakened and broadened diffraction peaks, indicating the clear refinement of grain size and amorphous transition. Moreover, there are not any other contaminates for the P-milling samples even under a mass ratio of 100:1, indicating that the P-milling method would not destroy the crystal structure of Si particles. Furthermore, the morphology logical of pristine Si and P-milling samples are observed via SEM. As can be seen in Figure 1b, pristine Si exhibits large primary particles with an average diameter of 1~5 μm. After the P-milling process, Si particles in Figure 1c–f exhibit irregular and agglomerated secondary particles consisting of nanoscale primary particles. Specifically, when the mass ratio increases to 50:1 and 100:1, obtained P-milling samples own the smallest average diameter of only ~50 nm.

**Figure 2:**
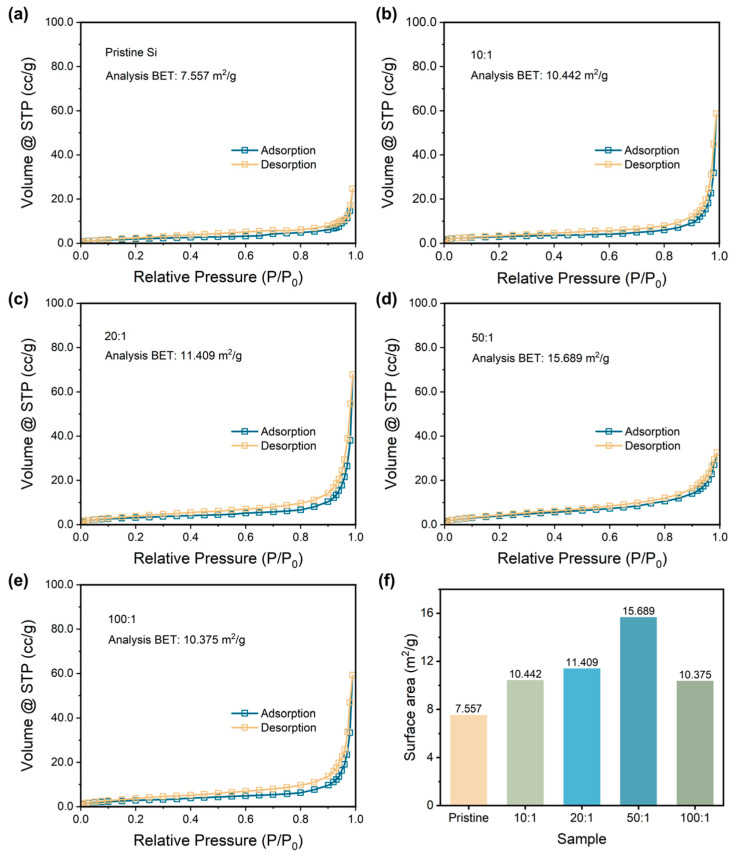
Nitrogen adsorption and desorption curves with analysis of the BET results of (**a**) pristine Si and p-milling samples with a mass ratio of (**b**) 10:1, (**c**) 20:1, (**d**) 50:1, and (**e**) 100:1. (**f**) Surface area for the pristine Si and P-milling samples.

Then, the specific surface areas are determined by applying the BET method to further compare the particle size of samples. Figure 2a–e exhibits the N_2_ adsorption and desorption curves of pristine Si and P-milling samples. All the samples show typical Type III isotherms without any hysteresis loop, indicating the samples are nonporous material [33]. Thus, the variation of the calculated specific surface area during the BET method depends on the particle size of the samples. Figure 2e lists the calculated BET value for all the samples. As the ratio increases, P-milling samples own the increased specific surface area; however, the specific surface area begins to decrease when the ball-to-material ratio is too high. Particularly, when a ball to material ratio is 50:1, the P-milling sample shows the largest specific surface area of 15.689 m^2^ g^−1^, which is more than two times than that of the pristine Si. The energy in the ball milling process increases with the ball-to-material ratio from 50:1 to 100:1, which intensifies the agglomeration, resulting in a decrease in specific surface area. Based on the above results, it can be concluded that pristine Si particles are successfully refined to nano-sized Si particles in a 4 h Ar-assisted P-milling process without other contaminations.

**Figure 3:**
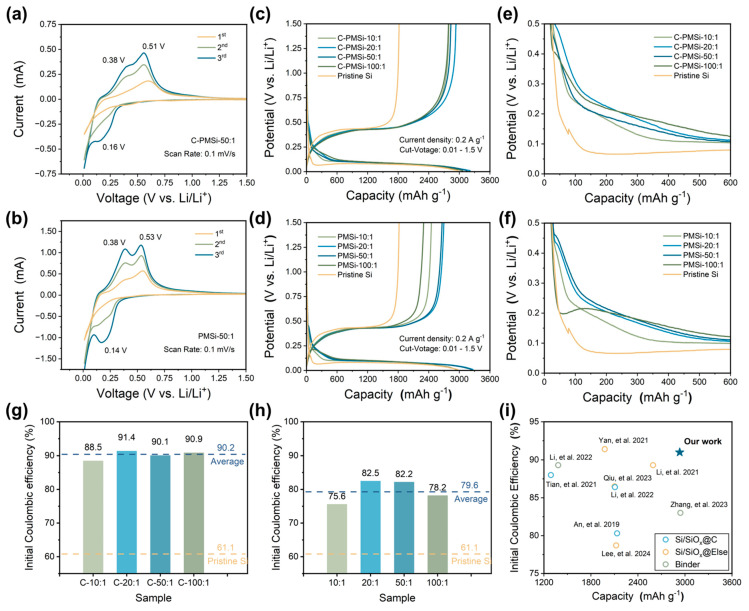
Initial CV curves of (**a**) C-PMSi-50:1 and (**b**) PMSi-50:1 anodes under a scan rate of 0.1 mV s-1. Initial galvanostatic charge–discharge curves of (**c**) C-PMSi and (**d**) PMSi anodes. Enlarged view of GCD curves of (**e**) C-PMSi and (**f**) PMSi anodes. Initial Coulombic efficiency of (**g**) C-PMSi and (**h**) PMSi anodes. (**i**) Comparisons of the ICE with recent literature reports [34,35,36,37,38,39,40,41,42].

Figure 3a,b displays the CV curves of PMSi-50:1 and C-PMSi-50:1 anodes in the first three cycles to investigate their lithiation/de-lithiation behavior. The wide cathodic peak in the initial cycle for the two samples corresponds to the formation of an SEI film on the Si anode. The C-PMSi-50:1 anode exhibits a more prominent broad peak than that of the PMSi-50:1 anode; moreover, the C-PMSi-50:1 anode shows weaker lithiation/de-lithiation redox intensity than that of the PMSi-50:1 anode, which can be attributed to the even formation of SEI film promoted via introducing CNTs, blocking the direct contact between Si particles and electrolyte during cycling. In the subsequent lithiation process, the reduction peak at ~0.16 V stands for the phase transition of Si to Li_x_Si, while the two oxidation peaks that occurred at ~0.38 and ~0.51 V in the de-lithiation processes are related to the two-step dealloying process of the Li_x_Si alloy to Si.

Figure 3c,d exhibits the initial galvanostatic charge–discharge (GCD) curves of pristine and P-milling anodes in half-cells under 0.2 A g^−1^. All the anodes exhibit similar GCD curves, indicating that the CNT networks would not be involved in the lithiation/de-lithiation process. Clearly, all the C-PMSi and PMSi anodes exhibit higher average initial discharge capacity of 3170.7 and 3145.3 mAh g^−1^ than 2972.5 mAh g^−1^ of the pristine Si. Compared with the pristine Si anode, C-PMSi and PMSi anodes own significantly increased discharge platforms and a smaller overpotential, indicating better electrochemical reversibility. However, C-PMSi anodes exhibit smaller discharge capacity than the PMSi anodes, which is due to the additional CNTs. Figure 3e,f displays the enlarged view of GCD curves for two electrodes, focusing the voltage window from 0 to 0.5 V of the early charge–discharge progress. Compared with the Si anode, C-PMSi and PMSi anodes own significantly increased discharge platform and a smaller overpotential, indicating better electrochemical reversibility.

Furthermore, the corresponding initial Coulombic efficiency (ICE) of C-PMSi and PMSi anodes is illustrated in Figure 3g and h. It can be seen that the electrochemical kinetics of Si anodes are observably enhanced by introducing CNTs and the P-milling method. Compared with pristine Si anodes, the average ICE level of PMSi anodes is enhanced from 61.1% to 79.6%, indicating the enhanced ion/electron diffusion rate in the nanoscale Si particles after P-milling. For the C-PMSi anodes, the ICE is dramatically enhanced with an average ICE of 90.2% via introducing the CNT network, implying the maximined transmission of ion/electron during the lithiation/de-lithiation process. In detail, ICEs of C-PMSi and PMSi anodes are dramatically enhanced by adjusting the mass ratio from 10:1 to 20:1 and start to decline when the mass ratio is raised from 20:1 to 100:1, which may be due to the low ion/electron transport efficiency caused by uneven size distribution and agglomeration. As shown in Figure 3i, compared with the recently reported Si-based anodes [34,35,36,37,38,39,40,41,42], the C-PMSi anodes exhibit not only high ICEs but also a high reversible capacity in the initial cycle with such high content of Si, indicating excellent reversibility and conductivity.

**Figure 4:**
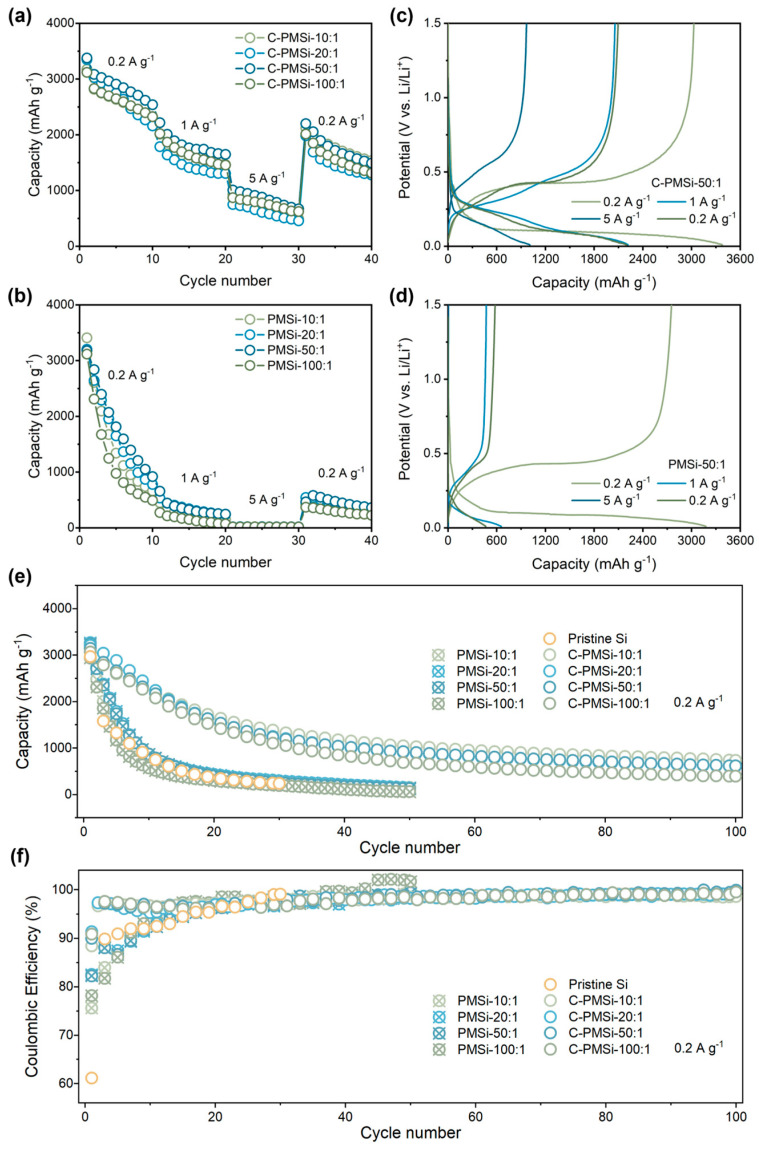
Rate capability of (**a**) C-PMSi and (**b**) PMSi anodes with the corresponding charge–discharge curves of (**c**) C-PMSi and (**d**) PMSi anodes. (**e**) Cyclic property and (**f**) Coulombic efficiency of PMSi, C-PMSi, and pristine anodes under 0.2 A g^−1^.

The rate capability of pristine and P-milling anodes are further tested under current densities increasing from 0.2 to 5 A g^−1^. As shown in Figure 4a,b, C-PMSi anodes present excellent high specific capacity than that of PMSi anodes. Specifically, C-PMSi-50:1 anodes can still offer a specific discharge capacity of ~1000 mAh g^−1^ at 5 A g^−1^, while PMSi anodes barely present any capacity. When the current density recovers back to 0.2 A g^−1^, the capacity of C-PMSi anodes is nearly four times higher than that of PMSi anodes. Moreover, the electrochemical behavior for C-PMSi-50:1 and PMSi-50:1 anodes under different current densities are discussed. The evolution of electrochemical behavior for C-PMSi-50:1 and PMSi-50:1 anodes under different current densities can be observed in Figure 4c,d, it can be seen that the C-PMSi-50:1 anode owns lower electrochemical overpotential and strong discharge depth under large current density, further indicating its enhanced conductivity and Li^+^ ion transfer rate.

Figure 4e,f shows the cyclic property and Coulombic efficiency (CE)-cycle curves of Pristine Si, C-PMSi, and PMSi anodes under 0.2 A g^−1^. It can be seen that the Si anode can hardly work in only 20 cycles. After being refined by P-milling, PMSi anodes present improved discharge capacity compared to that of a pristine Si anode; however, the improvement in capacity retention is finite because of the agglomeration of Si particles. C-PMSi anodes exhibit higher discharge capacity and better cyclic properties. The discharge capacity of C-PMSi anodes remained stable after ~30 cycles with an average reversible capacity of 615 mAh g^−1^ in the 100th cycle, while PMSi anodes deliver only below 300 mAh g^−1^ after 20 cycles. As shown in the CE-cycle curves, the CEs during the cycling of C-PMSi anodes are more stable than those of PMSi and pristine Si anodes, resulting in better capacity retention for C-PMSi anodes. The capacity reduction is effectively controlled by C-PMSi anodes due to the flexibility and strength of CNTs. Even so, all the C-PMSi anodes present low capacity retention (below 50%). Though it is acceptable for a pure Si anode, such performance is insufficient for practical application. Based on the above discussion, it can be concluded that the P-milling process and CNT additive can enhance the ICE of the anode by improving the electrochemical kinetics of the ion/electron transport process. Moreover, the CNTs’ conductive network helps to improve the capacity retention of PMSi anodes thanks to its high-strength tube structure.

**Figure 5:**
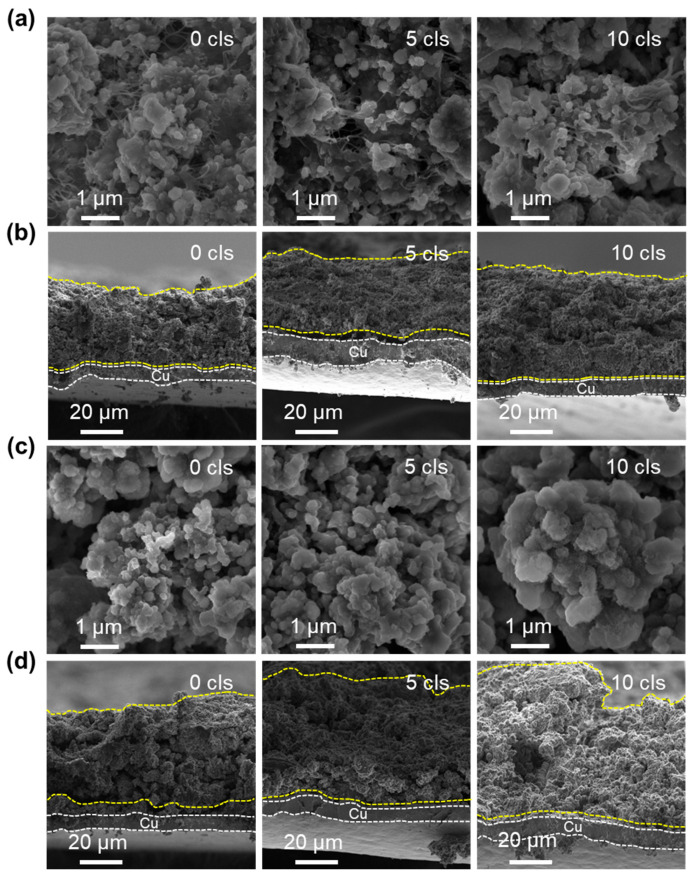
SEM images of the (**a**,**c**) surface and (**b**,**d**) cross-sections of C-PMSi-50:1 and PMSi-50:1 anodes before and after 5 and 10 cycles.

To deeply investigate the structural evolution of C-PMSi and PMSi anodes during continuous lithiation/de-lithiation processes, interfacial and cross-section structure of C-PMSi-50:1 and PMSi-50:1 anodes before and after 5th and 10th cycles were observed via SEM. Figure 5a exhibits that CNTs can be observed clearly among particles and anode components of the C-PMSi-50:1 anode, forming a uniform, conductive, and intensive connecting network, indicating that introducing CNTs would not destroy the structure of silicon anodes. The even distribution of the CNT network still remains distinct after the 5th and 10th cycles in the C-PMSi-50:1 anode, while Si particles remain at nanoscale size without clear growth and agglomeration. Conversely, such disconnection in the PMSi-50:1 anode results in a loss of electric contact and ultimately leads to a capacity reduction. As the cycles go on, the Si particles show clear agglomeration and growth of the PMSi-50:1 anode in Figure 5c; in particular, the 10th cycled sample shows micron-level Si particles.

Moreover, cross-sections of pristine and cycled C-PMSi-50:1 and PMSi-50:1 anodes are further compared. As shown in Figure 5b, the C-PMSi-50:1 anode exhibits stable and flat cross-section structures, indicating that the CNT network helps to maintain the anode structure away from collapse. As for the PMSi-50:1 anode, Si particles are on the verge of collapse after only 10 cycles in Figure 5d. The cycled PMSi-50:1 anode is hard to maintain the structure of by binders only and the Si particles tend to form a rough surface during repeating volume expansion with a high expansion rate of almost 100%. The 10th discharged C-PMSi-50:1 anode clearly exhibits suppressed volume expansion with a uniform thickness, revealing that CNT networks help to mitigate the electric disconnection caused by dramatic volume expansion of Si particles during cycling. Without the CNT network, the anode is hard to maintain its structure only by binders and the Si particles tend to form a rough surface during repeating volume expansion, resulting in inferior cyclic properties.

**Figure 6:**
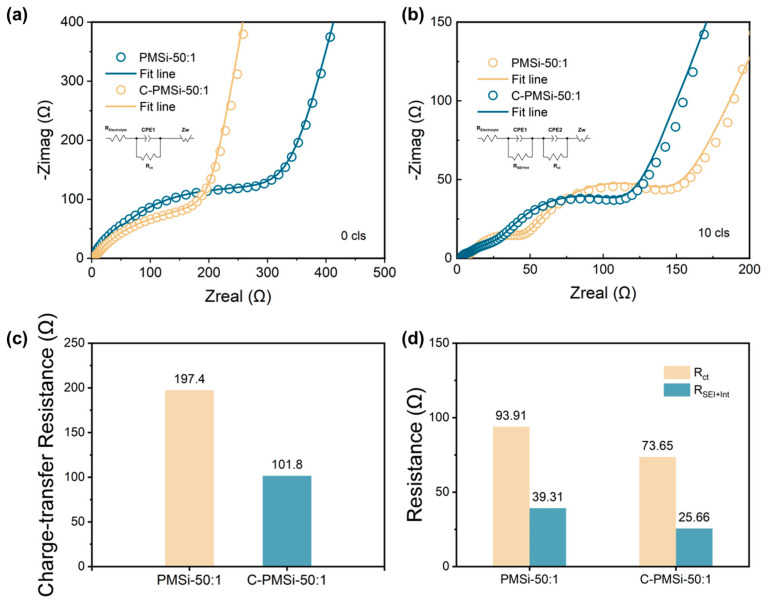
Nyquist plots of C-PMSi-50:1 and PMSi-50:1 anodes (**a**) before and (**b**) 10th discharge. Impedance parameters of C-PMSi-50:1 and PMSi-50:1 anodes (**c**) before and (**d**) 10th discharge.

The EIS was employed to evaluate the electronic conductivity for C-PMSi-50:1 and PMSi-50:1 anodes, which are presented in Figure 6a,b. Both anodes were measured before and after the 10th lithiation process with sufficient time (>6 h after finishing the lithiation process). It is exhibited that two electrodes own similar EIS plots. Before cycling, the plots consisted of one semicircle curve in the high-frequency area with a liner plot in the low-frequency area, while after the 10th lithiation, an additional semicircle curve appeared in the high-frequency plot. The EIS curves are fitted via the standard equivalent circuit model and the impedance parameters are illustrated in Figure 6c,d, consisting of electrolyte resistance (R_Eletrolyte_), surface film capacitance (R_SEI+Int_), charge-transfer resistance at the electrode, and electrolyte interface (R_ct_). The R_ct_ reflecting the low intrinsic electron conductivity of pure Si and the R_SEI+Int_ can be used to compare the formation and thickness of the SEI layer. Clearly, the C-PMSi-50:1 anode exhibits lower R_ct_ before and after cycling than PMSi-50:1, benefiting from the conductive CNTs network in anodes. After the 10th cycle, the R_SEI+Int_ of C-PMSi-50:1 and PMSi-50:1 is 25.66 Ω and the R_ct_ is 39.31 Ω, representing that PMSi-50:1 owns thicker SEI after repeating lithiation/de-lithiation without CNT networks maintaining the Si particles. Even by adding conductive C45 and CNTs to manufacture an electrode, C-PMSi-50:1 still represents a large R_ct_. By compositing conductive material with Si, such as Sn, C, Cr, etc., would improve the conductivity of the Si-based composite. The formation of the SEI layer in a Si-based anode basically depends on the component of the electrolyte and the volume change in the Si-based anode. For better stability, the structural design of Si particles is essential.

**Figure 7:**
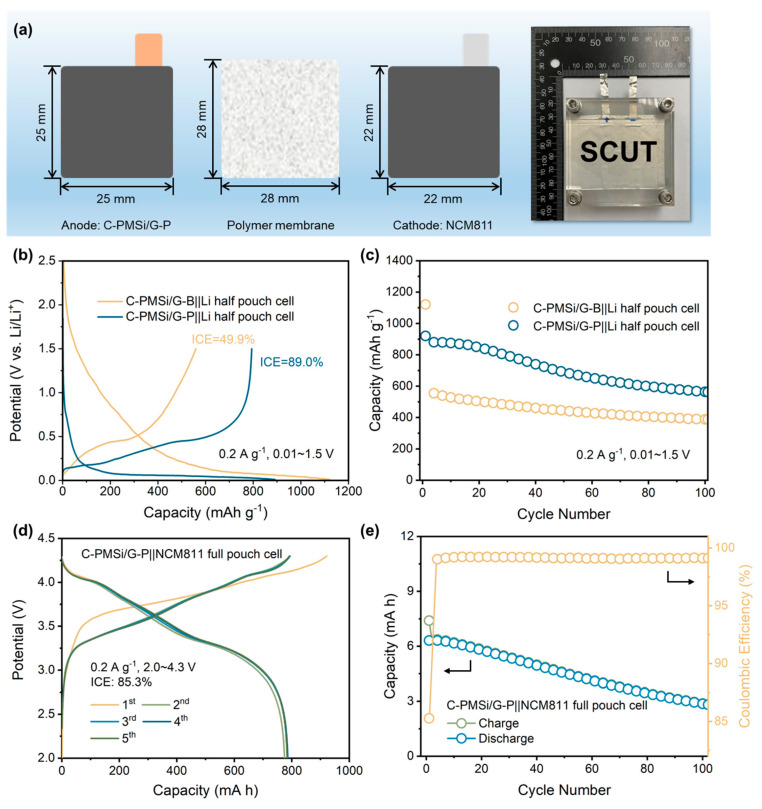
(**a**) Structure diagram of a pouch cell. (**b**) Initial galvanostatic charge–discharge curves and (**c**) cyclic property of C-PMSi/G-B||Li and C-PMSi/G-P||Li half-cell. (**d**) Initial galvanostatic charge–discharge curves and (**e**) cyclic property of the C-PMSi/G-P||NCM811 full pouch cell.

For deeply promoting the practical application of Si anodes, both PMSi/G||Li half and PMSi/G||NCM811 full pouch cell (Figure 7a) are manufactured. Initial galvanostatic charge–discharge curves and cyclic properties of C-PMSi/G||Li prepared by two different blending processes are measured under 0.2 A g^−1^. As displayed in Figure 7b, the C-PMSi/G-P anode could deliver a high ICE of 89.0%, which is much higher than 49.9% of the C-PMSi/G-B anode, further confirming that the P-milling process could be applied as an efficient mixing method without destroying morphology of particles. Moreover, Figure 7c exhibits that the C-PMSi/G-P anode could deliver a higher 100th discharge capacity with the capacity retention of 891.7 mAh g^−1^ and 67.1%, while those of C-PMSi/G-B are only 388.4 mAh g^−1^ and 34.6%. For the C-PMSi/G-P anode, the capacity retention is improved by blending with graphite. Usually, the commercial anodes contain less than 10 wt. % of Si-based material for avoiding its volume expansion during lithiation/de-lithiation. Thus, the C-PMSi/G-P anode would be more stable by adjusting the amount of PMSi. To explore its practical ability, the C-PMSi/G-P anode and NCM811 cathode are assembled as the full pouch cell. As displayed in Figure 7d, the C-PMSi/G||NCM811 full pouch cell exhibits a high ICE of 85.3% with the initial lithiation capacity of 7.4 mA h, i.e., 921.7 mAh g^−1^ based on the mass loading of the C-PMSi/G anode, operating in 2.0~4.4 V at 0.2 A g^−1^. After 100 cycles, Figure 7e illustrates that the manufactured full-cell remains at a high 100th de-lithiation capacity of 356.7 mAh g^−1^ with a stable Coulombic efficiency with an average value of 99.2%, indicating its good electrochemical irreversibility.

## 4. Conclusions

In summary, we prepared the stable C-PMSi anodes with the uniform CNT conductive network via the highly effective P-milling method. The mass ratio of balls to the material of the P-milling method is discussed in detail. Benefiting from the firm structure and continuous ion/electron transport pathways of the CNT network and nano-sized Si particles, C-PMSi anodes demonstrate high average ICEs of 90.2% with a reversible average capacity of 587.7 mAh g^−1^ at 0.2 A g^−1^ after 100 cycles. Moreover, the PMSi||Li assembled with graphite-blended C-PMSi/G-P anode exhibits a high ICE of 89.0% and a reversible capacity of 598.3 mAh g^−1^ at 0.2 A g^−1^ after 100 cycles. The pouch-type C-PMSi/G-P||NCM811 full-cell could also deliver a high ICE of 85.3% with the reversible capacity of 356.7 mAh g^−1^ at 0.2 A g^−1^ after 100 cycles, together with the simple and efficient P-milling method. Our work may widen the applications of the DBDP-assisted milling process and supply a new sight of designing Si-based anode materials for high energy density Li-ion batteries and devices. However, the electrode design still needs to be improved by adjusting the components in the electrode, especially for CNTs. On the other hand, the structural design and composited with conductive material is essential for Si particles.

## Data Availability

The data that support the findings of this study are available from the corresponding author upon reasonable request.

## References

[1] Tarascon J.-M., Armand M. (2001). Issues and Challenges Facing Rechargeable Lithium Batteries. Nature.

[2] Armand M., Tarascon J.-M. (2008). Building Better Batteries. Nature.

[3] Dunn B., Kamath H., Tarascon J.-M. (2011). Electrical Energy Storage for the Grid: A Battery of Choices. Science.

[4] Li M., Lu J., Chen Z., Amine K. (2018). 30 Years of Lithium-Ion Batteries. Adv. Mater..

[5] Nitta N., Wu F., Lee J.T., Yushin G. (2015). Li-Ion Battery Materials: Present and Future. Mater. Today.

[6] Li J., Fleetwood J., Hawley W.B., Kays W. (2022). From Materials to Cell: State-of-the-Art and Prospective Technologies for Lithium-Ion Battery Electrode Processing. Chem. Rev..

[7] Bryntesen S.N., Strømman A.H., Tolstorebrov I., Shearing P.R., Lamb J.J., Stokke Burheim O. (2021). Opportunities for the State-of-the-Art Production of LIB Electrodes—A Review. Energies.

[8] Degen F., Winter M., Bendig D., Tübke J. (2023). Energy Consumption of Current and Future Production of Lithium-Ion and Post Lithium-Ion Battery Cells. Nat. Energy.

[9] Ge M., Cao C., Biesold G.M., Sewell C.D., Hao S.-M., Huang J., Zhang W., Lai Y., Lin Z. (2021). Recent Advances in Silicon-Based Electrodes: From Fundamental Research toward Practical Applications. Adv. Mater..

[10] Qi Y., Wang G., Li S., Liu T., Qiu J., Li H. (2020). Recent Progress of Structural Designs of Silicon for Performance-Enhanced Lithium-Ion Batteries. Chem. Eng. J..

[11] Obrovac M.N., Krause L.J. (2006). Reversible Cycling of Crystalline Silicon Powder. J. Electrochem. Soc..

[12] Choi J.W., Aurbach D. (2016). Promise and Reality of Post-Lithium-Ion Batteries with High Energy Densities. Nat. Rev. Mater..

[13] Liang B., Liu Y., Xu Y. (2014). Silicon-Based Materials as High Capacity Anodes for next Generation Lithium Ion Batteries. J. Power Sources.

[14] Tzeng Y., Jhan C.-Y., Wu Y.-C., Chen G.-Y., Chiu K.-M., Guu S.Y.-E. (2022). High-ICE and High-Capacity Retention Silicon-Based Anode for Lithium-Ion Battery. Nanomaterials.

[15] Chan C.K., Peng H., Liu G., McIlwrath K., Zhang X.F., Huggins R.A., Cui Y. (2008). High-Performance Lithium Battery Anodes Using Silicon Nanowires. Nat. Nanotechnol..

[16] McDowell M.T., Lee S.W., Ryu I., Wu H., Nix W.D., Choi J.W., Cui Y. (2011). Novel Size and Surface Oxide Effects in Silicon Nanowires as Lithium Battery Anodes. Nano Lett..

[17] Keller C., Desrues A., Karuppiah S., Martin E., Alper J.P., Boismain F., Villevieille C., Herlin-Boime N., Haon C., Chenevier P. (2021). Effect of Size and Shape on Electrochemical Performance of Nano-Silicon-Based Lithium Battery. Nanomaterials.

[18] Ma C., Wang Z., Zhao Y., Li Y., Shi J. (2020). A Novel Raspberry-like Yolk-Shell Structured Si/C Micro/Nano-Spheres as High-Performance Anode Materials for Lithium-Ion Batteries. J. Alloys Compd..

[19] Zhou J., Qian T., Wang M., Xu N., Zhang Q., Li Q., Yan C. (2016). Core–Shell Coating Silicon Anode Interfaces with Coordination Complex for Stable Lithium-Ion Batteries. ACS Appl. Mater. Interfaces.

[20] Han N., Li J., Wang X., Zhang C., Liu G., Li X., Qu J., Peng Z., Zhu X., Zhang L. (2021). Flexible Carbon Nanotubes Confined Yolk-Shelled Silicon-Based Anode with Superior Conductivity for Lithium Storage. Nanomaterials.

[21] Li X., Gu M., Hu S., Kennard R., Yan P., Chen X., Wang C., Sailor M.J., Zhang J.-G., Liu J. (2014). Mesoporous Silicon Sponge as an Anti-Pulverization Structure for High-Performance Lithium-Ion Battery Anodes. Nat. Commun..

[22] Li X., Yan P., Xiao X., Woo J.H., Wang C., Liu J., Zhang J.-G. (2017). Design of Porous Si/C–Graphite Electrodes with Long Cycle Stability and Controlled Swelling. Energy Environ. Sci..

[23] Magazzù A., Marcuello C. (2023). Investigation of Soft Matter Nanomechanics by Atomic Force Microscopy and Optical Tweezers: A Comprehensive Review. Nanomaterials.

[24] Grigoriev S., Metel A., Mustafaev E., Melnik Y., Volosova M. (2023). Micro End Mill Capability Improvement Due to Processing by Fast Argon Atoms and Deposition of Wear-Resistant Coating. Metals.

[25] Cheng D., Liu J., Li X., Hu R., Zeng M., Yang L., Zhu M. (2017). A Highly Stable (SnOx-Sn)@few Layered Graphene Composite Anode of Sodium-Ion Batteries Synthesized by Oxygen Plasma Assisted Milling. J. Power Sources.

[26] Dai L.Y., Cao B., Zhu M. (2006). Comparison on Refinement of Iron Powder by Ball Milling Assisted by Different External Fields. Acta Metall. Sin. Engl. Lett..

[27] Meng F., Hu R., Chen Z., Tan L., Lan X., Yuan B. (2021). Plasma Assisted Synthesis of LiNi_0.6_Co_0.2_Mn_0.2_O_2_ Cathode Materials with Good Cyclic Stability at Subzero Temperatures. J. Energy Chem..

[28] Sun W., Hu R., Zhang H., Wang Y., Yang L., Liu J., Zhu M. (2016). A Long-Life Nano-Silicon Anode for Lithium Ion Batteries: Supporting of Graphene Nanosheets Exfoliated from Expanded Graphite by Plasma-Assisted Milling. Electrochimica Acta.

[29] Li X., Sun X., Hu X., Fan F., Cai S., Zheng C., Stucky G.D. (2020). Review on Comprehending and Enhancing the Initial Coulombic Efficiency of Anode Materials in Lithium-Ion/Sodium-Ion Batteries. Nano Energy.

[30] Meng F., Zhang H., Xiong X., Li X., Wu R., Han Q., Qin B., Yuan B., Hu R. (2024). Revealing the Subzero-Temperature Electrochemical Kinetics Behaviors in Ni-Rich Cathode. Small.

[31] He Z., Xiao Z., Yue H., Jiang Y., Zhao M., Zhu Y., Yu C., Zhu Z., Lu F., Jiang H. (2023). Single-Walled Carbon Nanotube Film as an Efficient Conductive Network for Si-Based Anodes. Adv. Funct. Mater..

[32] Park S.-H., King P.J., Tian R., Boland C.S., Coelho J., Zhang C., McBean P., McEvoy N., Kremer M.P., Daly D. (2019). High Areal Capacity Battery Electrodes Enabled by Segregated Nanotube Networks. Nat. Energy.

[33] Thommes M., Kaneko K., Neimark A.V., Olivier J.P., Rodriguez-Reinoso F., Rouquerol J., Sing K.S.W. (2015). Physisorption of Gases, with Special Reference to the Evaluation of Surface Area and Pore Size Distribution (IUPAC Technical Report). Pure Appl. Chem..

[34] An W., Gao B., Mei S., Xiang B., Fu J., Wang L., Zhang Q., Chu P.K., Huo K. (2019). Scalable Synthesis of Ant-Nest-like Bulk Porous Silicon for High-Performance Lithium-Ion Battery Anodes. Nat. Commun..

[35] Qiu Z., Wu A., Yu W., Li A., Dong X., Huang H. (2023). Si-TiSi2 Clusters Eutectic Nanoparticles as High Initial Coulombic Efficiency Anodes for Lithium-Ion Batteries. Electrochim. Acta.

[36] Lee I.-H., Jin Y., Jang H.-S., Whang D. (2024). Enhancing the Stability and Initial Coulombic Efficiency of Silicon Anodes through Coating with Glassy ZIF-4. Nanomaterials.

[37] Zhang Q., Yu Y., Li H., Zhang F., Liu Y. (2023). Effect of Silicone Segment and Imidization of Polyimide-Based Binder on the Electrochemical Performances of High Silicon Content Anode for Lithium-Ion Batteries. Electrochim. Acta.

[38] Yan Y., He Y.-S., Zhao X., Zhao W., Ma Z.-F., Yang X. (2021). Regulating Adhesion of Solid-Electrolyte Interphase to Silicon via Covalent Bonding Strategy towards High Coulombic-Efficiency Anodes. Nano Energy.

[39] Li Z., Tang W., Yang Y., Lai G., Lin Z., Xiao H., Qiu J., Wei X., Wu S., Lin Z. (2022). Engineering Prelithiation of Polyacrylic Acid Binder: A Universal Strategy to Boost Initial Coulombic Efficiency for High-Areal-Capacity Si-Based Anodes. Adv. Funct. Mater..

[40] Li L., Deng J., Wang L., Wang C., Hu Y.H. (2021). Boron-Doped and Carbon-Controlled Porous Si/C Anode for High-Performance Lithium-Ion Batteries. ACS Appl. Energy Mater..

[41] Li S., Shi X.-Y., Tang Z.-P., Li D.-X., Zhang Y.-C., Xiao Y., Song Y., Zheng Z., Zhong Y.-J., Wu Z.-G. (2022). Simultaneous Enhancement of Initial Coulombic Efficiency and Cycling Performance of Silicon-Based Anode Materials for Lithium-Ion Batteries. Appl. Surf. Sci..

[42] Tian H., Tian H., Yang W., Zhang F., Yang W., Zhang Q., Wang Y., Liu J., Silva S.R.P., Liu H. (2021). Stable Hollow-Structured Silicon Suboxide-Based Anodes toward High-Performance Lithium-Ion Batteries. Adv. Funct. Mater..

